# A dual self-attentive transformer U-Net model for precise pancreatic segmentation and fat fraction estimation

**DOI:** 10.1186/s12880-025-01852-5

**Published:** 2025-08-04

**Authors:** Ashok Shanmugam, Prianka Ramachandran Radhabai, Kavitha KVN, Agbotiname Lucky Imoize

**Affiliations:** 1 Department of Electronics and Communication Engineering, Vel Tech Multi Tech Dr. Rangarajan Dr. Sakunthala Engineering College, Chennai, Tamil Nadu India; 2 Department of CSE, Manipal Institute of Technology, Bangalore, Karnataka India; 3 Department of Communication Engineering, School of Electronics Engineering, Vellore Institute of Technology, Vellore, Tamil Nadu India; 4 Department of Electrical and Electronics Engineering, Faculty of Engineering, University of Lagos, Akoka, Lagos 100213 Nigeria

**Keywords:** Computed tomography (CT), Pancreatic segmentation, Transformer Unet model, Attention mechanism, Fat fraction estimation

## Abstract

Accurately segmenting the pancreas from abdominal computed tomography (CT) images is crucial for detecting and managing pancreatic diseases, such as diabetes and tumors. Type 2 diabetes and metabolic syndrome are associated with pancreatic fat accumulation. Calculating the fat fraction aids in the investigation of β-cell malfunction and insulin resistance. The most widely used pancreas segmentation technique is a U-shaped network based on deep convolutional neural networks (DCNNs). They struggle to capture long-range biases in an image because they rely on local receptive fields. This research proposes a novel dual Self-attentive Transformer Unet (DSTUnet) model for accurate pancreatic segmentation, addressing this problem. This model incorporates dual self-attention Swin transformers on both the encoder and decoder sides to facilitate global context extraction and refine candidate regions. After segmenting the pancreas using a DSTUnet, a histogram analysis is used to estimate the fat fraction. The suggested method demonstrated excellent performance on the standard dataset, achieving a DSC of 93.7% and an HD of 2.7 mm. The average volume of the pancreas was 92.42, and its fat volume fraction (FVF) was 13.37%.

## Introduction

Pancreatic diseases cause significant morbidity and mortality and are important subjects for clinical research [1–3]. Pancreatic cancer is currently the 7th leading cause of mortality globally [4]. Imaging biomarkers representing fundamental structural variations within the pancreas have been identified through radiological studies of the organ. These biomarkers can be crucial for the early detection of metabolic illnesses, including diabetes and pancreatic cancers [5]. Because of late diagnosis and a miserable prediction, just 25% of patients live for a year, and only 2% to 12% live for 5 years. Fat deposition in pancreatic tissue is referred to as pancreatic fat content, pancreatic steatosis, or fatty pancreas. As pancreatic fat content increases with the progression of metabolic diseases, pancreatic dysfunction, and diabetes, it has become a significant focus of both clinical and research attention. The pancreas plays a crucial role in diabetes because it produces the hormone insulin, which is essential for regulating blood glucose levels. Within the pancreatic islets, specialized cells called β-cells release insulin. Pancreatic steatosis, or excessive fat deposition in the pancreas, impairs β-cell function in diabetes, particularly type 2, by reducing insulin production and contributing to insulin resistance. This results in a decrease in blood sugar levels and an increase in diabetes. Therefore, understanding and accurately measuring pancreatic fat is crucial for the early detection and treatment of diabetes and related metabolic disorders.

Moreover, the most accurate assessment of pancreatic fat would potentially be obtained through histological examination of pancreatic tissue [6, 7]. However, this is unlikely to be a therapeutically valuable tool for assessing pancreatic fat due to the inherent risks of pancreatic biopsy and sampling inaccuracy [6]. Several cross-sectional imaging approaches, including computed tomography (CT) and magnetic resonance imaging (MRI), have been investigated to identify FP to address this problem [7, 8]. Since the pancreas is a small, asymmetrical organ encircled by fat, intestines, and other abdominal tissues, accurate segmentation is necessary for pancreatic fat quantification. If the pancreas is not accurately segmented, contamination by surrounding tissue may result in inaccurate fat estimates. This highlights the importance of improving pancreatic segmentation. Nevertheless, a significant obstacle to three-dimensional segmentation of the pancreas is the lack of proven and repeatable technology [9, 10]. The widely utilized imaging technique for the preliminary assessment of pancreatic masses is computed tomography (CT) [11–13]. Nonetheless, there are three difficulties when it comes to segmenting pancreatic tumors on CT scans. First, given the pancreatic mass density, solid elements are challenging to distinguish from pancreatic parenchyma, while Fluid-containing elements are jumbled with pancreatic canals or cystic ducts. Second, the dimensions, silhouette, texture, and contrast of pancreatic masses vary greatly. Thirdly, there are various types of pancreatic masses [14].

Recent research has demonstrated that artificial intelligence (AI) can equal or even outperform human professionals on various medical image processing tasks, despite experienced radiologists finding it challenging to distinguish between pancreatic and non-contrast CT [15–17]. The rapid development of CNNs has made AI useful in medical image processing [18, 19]. Current medical image segmentation techniques mostly rely on U-shaped fully convolutional networks (FCN) [20, 21]. U-Net and its variations are frequently used to segment medical images in various contexts. They include a symmetric encoder-decoder network with skip connections for enhanced detail representation [13]. CNN-based U-shaped networks struggle to capture long-range semantic linkages because of their inability to perceive global contextual information. They can perform feature mining of local data [22].

Transformer was initially developed for natural language processing (NLP) tasks that involve sequence-to-sequence modeling. However, the transformer’s capacity to describe long-range relationships makes it appropriate for computer vision tasks [23]. However, the transformer might not be the best option for analyzing high-dimensional data, such as CT images, due to the network’s numerous parameters. Medical image segmentation using the original transformer network may result in sluggish training and decreased efficacy. Additionally, various types of noise will be generated when medical images are acquired and transmitted. Networks will therefore extract features from noisy systems, producing imprecise findings. Hence, designing new, fresh transformer models for precisely segmenting the pancreas and generalized pancreatic masses is crucial.

### Motivation

Accurate segmentation of the pancreas and pancreatic tissue on CT scans is crucial for the early detection and treatment of pancreatic disorders, including diabetes and cancer. However, segmentation is challenging due to the pancreas’s intricate structure, its limited size, and the presence of neighboring tissues. While U-Net and other traditional convolutional neural networks (CNNs) excel at capturing local features, they struggle to model global context and long-range connections, which are essential for accurate segmentation in complex anatomical regions.

Transformers are robust self-monitoring algorithms that can capture global relationships; they were initially developed for natural language processing. However, the high processing costs, slow training, and susceptibility to noise inherent in medical imaging limit their direct application to high-dimensional medical images such as CT scans. This study addresses the above issues by introducing a novel dual self-attentive transformer (DSAT) architecture that combines the strengths of CNNs for local spatial detail with transformers for enhanced global context understanding.

This research aims to enhance segmentation accuracy by integrating dual self-attentive transformers into the encoder and decoder, particularly in complex anatomical regions where traditional U-Nets are insufficient. The proposed approach extracts low-level spatial characteristics for in-depth local representation using a convolutional neural network (CNN) encoder. The DSAT encoder improves global feature understanding by enhancing feature interactions through self-attention. Additionally, the DSAT decoder utilizes Masked Cross-attention refinement to ensure accuracy in per-pixel segmentation. Lastly, the CNN decoder preserves attention-enhanced feature maps while restoring spatial details. The following is the list of primary contributions of this research work:

- This work suggests a unique architecture called DSTUnet to increase the accuracy of pancreatic segmentation, which is essential for evaluating pancreatic fat and associated metabolic disorders. Dual Self-Attentive Swin Transformers (DSAT) are incorporated into the encoder and decoder phases of DSTUnet, forming a U-shaped network. Swin Transformers allow hierarchical attention mechanisms that may efficiently simulate long-range dependencies within medical images, in contrast to conventional deep convolutional neural networks (DCNNs). DCNNs are constrained by local receptive fields and struggle to capture global context.
- This work introduces a Dual Self-Attention mechanism into the Transformer model for concurrently capturing features from both the frequency and spatial domains. These features enable the model to reliably and accurately distinguish the pancreas from surrounding tissue, even in the presence of slight variations or anatomical heterogeneity.
- This work incorporates a Granular Attention Refinement (GAR) method into the Transformer decoder to improve significantly the segmentation of the pancreas, especially in identifying smaller and more anatomically subtle parts. It employs a coarse-to-fine refinement technique, where an initial rough segmentation mask guides a more precise and targeted refinement process. In particular, the DSAT decoder features a mask attention module that enables the model to selectively focus on the foreground area based on its previous coarse predictions.
- One of its key contributions is that this study incorporates histogram-based analysis to measure pancreatic fat accumulation, a key indicator for metabolic diseases, including type 2 diabetes and metabolic syndrome. Histogram analysis provides a non-invasive method for monitoring fat content by detecting variations in voxel intensity that correlate with fatty regions versus normal pancreatic parenchyma.

The remainder of the paper is structured as follows: Section "Literature survey" reviews the recent literature on pancreatic segmentation and fat fraction estimation. Section "Proposed method" gives a detailed description of the proposed model. Section "Results and discussion" validates the effectiveness of the proposed model using simulated results. Section "Conclusion" presents a conclusion and outlines the scope of future research.

## Literature survey

Although anatomical heterogeneity and low tissue differentiation make accurate pancreatic segmentation challenging, it is essential for clinical applications such as fat assessment and tumor detection. Recent research has investigated CNNs and Transformer-based models to enhance segmentation performance. This review outlines the main developments and their limitations, promoting the proposed DSTUnet.

Zhang et al. [24] presented PanSegNet, a novel pancreatic segmentation technique that combines the advantages of nnUNet and a Transformer model with a novel linear attention unit, allowing for volume calculation. A linear self-attention layer was the bottleneck of the PanSegNet structure, and it was adapted from a typical self-attention method. The PanSegNet structure was developed using nnUNet as its backbone, and it employs an encoder–decoder–style segmentation. The semantic features were extracted throughout the encoding process. The decoding method created a segmentation mask using the retrieved features and hierarchical structure.

Qu et al. [25] presented a transformer-guided progressive fusion network (TGPFN) that utilized the universal depiction acquired by the transformer to compensate for longer-range dependencies that were missed by convolutional processes at various dimensions. The foundation of TGPFN was a hierarchically fused network topology, in which the transformer branches and CNN extracted data independently in the encoder before gradually fusing the small-scale and large-scale features in the decoder. Additionally, a collaborative attention mechanism was implemented to capture channel dependencies and establish a transformer-based direction flow, ensuring feature consistency and facilitating the efficient integration of data from the two branches. Cao et al. [26] developed a pancreatic cancer detection system using AI (PANDA) to accurately identify and categorize pancreatic lesions on non-contrast CT scans.

Yang et al. [27] investigated the estimation of the Fat Density Ratio from specimens and the Fat Deposition in Pancreas using magnetic resonance spectroscopy (MRS) and Iterative Decomposition with Echo Asymmetry and Least-Squares estimation (IDEAL) in MRI. They developed a unique DCNN that resembled the MR-opsy approach for determining the pancreas volume and Fat Deposition in the Pancreas from abdominal MRI. Segmenting micro-organ elements, such as the pancreas, using DCNN was challenging, as the shape under MRI was diverse due to contamination from organ boundaries.

 Li et al. [28] developed a 3D full CNN with three temperature-assisted units: balance temperature loss, rigid temperature optimizer, and soft temperature indicator for jointly segmenting both the pancreas and tumors and in particular, balancing temperature loss aimed to increase tumor segmentation accuracy without sacrificing pancreatic information through the dynamic adjustment of the learning opinions between tumors and the pancreas. to adaptively avoid local optima, a rigid temperature optimizer was suggested, which accepted non-improving steps probabilistically. Additionally, a soft temperature indicator was intended to automatically steer the model into a refinement stage, and the network leans towards solidity to further enhance the segmentation outcomes.

Yao et al. [29] proposed a transferred DenseSE-Mask R-CNN (TDSMask R-CNN) for segmentation using Dense and SE units. The multi-scale characteristics approach was chosen to address the large degree of diversity in pancreatic tumors. This network could use an attention mechanism to learn complementary information from various modalities (PET/MR) images to identify pancreatic tumor locations in multiple domains. Consequently, it was possible to suppress the extraneous information needed to segment the tumor area and obtain low false positives. To mitigate the limited number of label samples and minimize network overfitting, the precise tumor location from the PET image was also sent to the MRI training model for guiding the Dense-SE network’s learning.

Zhang et al. [30] proposed a new segmentation model called nnTransfer (nonisomorphic transfer learning) net. It utilized a Self-supervised generative framework to enable the model to learn image features from unlabeled input. Common attributes that direct the segmentation task were extracted using a self-supervised learning approach from a significant amount of unlabeled abdominal CT image data. The Self-guided learning unit established the segmentation weights while the segmentation component extracted the pancreatic tissue. Additionally, an end-to-end pancreatic fat quantification was accomplished using a histogram examination with local thresholding.

According to this investigation, pancreatic segmentation is essential for estimating the fat fraction. Segmentation errors directly impact the estimation of the fat fraction. However, pancreatic segmentation poses several challenges due to the organ’s complexity and patient-specific variations. Feature extraction and border identification are enhanced by sophisticated deep learning models (e.g., DCNN [25], PanSegNet [24], TDSMask R-CNN [29], and nnTransfer [30]). Being an extended and extremely changeable organ, the pancreas must be distinguished from surrounding tissues using global context awareness. The CNN-based architectures employed by most current models, such as DCNN, PanSegNet, and TDSMask R-CNN, are effective at capturing local features but struggle with long-range dependencies. Non-isomorphic transfer learning (nnTransfer) has several drawbacks, despite its intention to improve segmentation performance by utilizing information from other datasets. The pre-trained models used in non-isomorphic transfer learning might not generalize successfully if there are substantial differences between the source and target domains. This suggests that a more sophisticated feature aggregation technique is required; feature transfer alone is insufficient. Alternatively, self-attention techniques (such as Transformers) can enhance feature aggregation over vast spatial regions. These points encourage us to introduce more advanced Dual Self-attention mechanisms in the Unet model to improve pancreatic cancer segmentation. The existing literature reviewed is summarized in Table 1.

**Table 1:** Summary of literature

Reference	Model/method	Advantages	Limitation
Zhang et al. [24]	PanSegNet (nnUNet + Transformer + Linear Attention)	Integrates the advantages of CNN and Transformer; volume estimation is supported.	Because of a linear attention bottleneck, long-range context modeling is limited.
Qu et al. [25]	TGPFN (Transformer-Guided Progressive Fusion Network)	Uses collective attention and captures multi-scale features.	Fusion is not entirely optimized for small structures due to its complex nature.
Cao et al. [26]	PANDA (AI system for lesion detection)	Prioritize the classification of lesions; high diagnostic precision	Used non-contrast CT; not segmentation-specific
Yang et al. [27]	MRS + IDEAL with DCNN	Allows for fat estimate; influenced by MR-opsy	Struggles with organ boundary contamination issues and inconsistent segmentation
Li et al. [28]	3D CNN with Temperature-Assisted Units	Used adaptive optimization that strikes a balance between tumor and pancreatic learning	Complex parameter changes and training
Yao et al. [29]	TDSMask R-CNN (PET/MRI with SE-Dense)	Learning in several modalities lowers false positives	It mostly depends on PET, which is limited in environments with limited resources.
Zhang et al. [30]	nnTransfer (Nonisomorphic Transfer Learning)	Self-supervised learning and unlabeled data	Problems with domain mismatch, variations in segmentation accuracy

The suggested DSTUnet successfully addresses the main shortcomings of the current approaches. In contrast to CNN-based models, which struggle with long-range dependencies, DSTUnet employs dual self-supervision to capture the global context. Additionally, it improves segmentation accuracy by paying granular attention to fine anatomical elements. In contrast to models that rely on transfer learning or multi-modal inputs, DSTUnet operates consistently with single-modality MRI. It closes a significant clinical gap by introducing histogram analysis for accurate assessment of pancreatic fat.

By combining CNNs with Swin transformers, the current literature has explored several architectures; our proposed DSTUnet model offers several significant innovations. Both the encoder and decoder used dual self-attenuating transformers (DSAT). DSTUnet employs specific self-attentive transformer modules in both the encoder and decoder paths, which contrasts with many previous studies that only utilize attention modules or transformers in the encoder. With this dual approach, we can simultaneously enhance fine-grained spatial feature reconstruction and global context extraction. This is required for accurate segmentation of small and intricate structures such as the pancreas. In addition, to the best of our knowledge, previous Swin-based segmentation models have not frequently addressed the dual self-monitoring mechanism that simultaneously records interactions in the spatial and frequency domains. The Granular Attention Refining (GAR) method incorporated into the DSTUnet decoder improves segmentation by performing coarse-to-fine refinement. With the help of trainable queries and a multi-head cross-attention (MCA) mechanism, the proposed DSAT decoder allows the network to focus on spatially significant areas adaptively. This represents a considerable improvement over the basic upsampling or skip connections commonly used in many current CNN-transformer hybrids.

## Proposed method

**Fig. 1:**
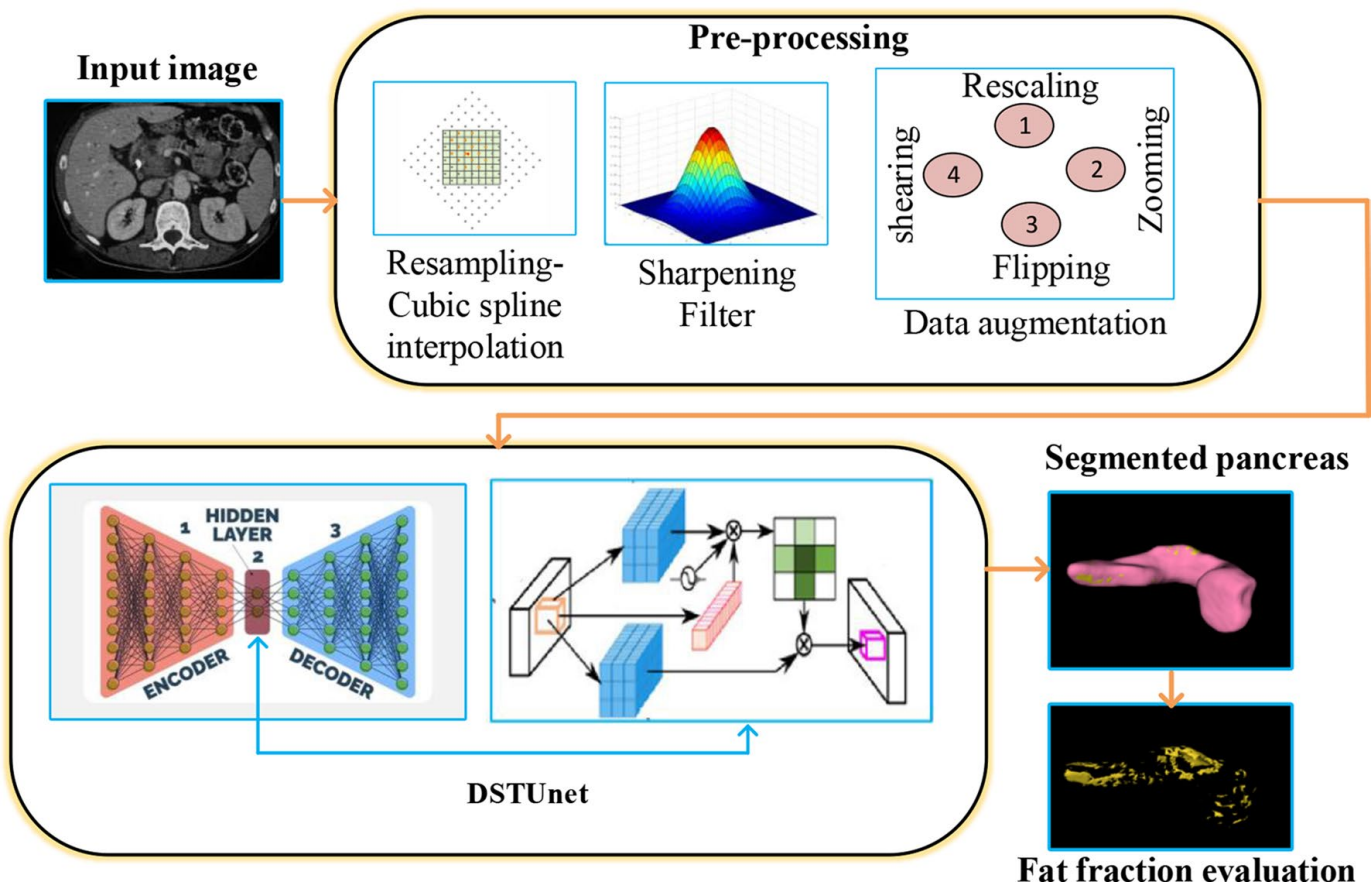
Overall flow of the proposed method

The proposed methodology provides an efficient approach for automatically segmenting the pancreas in abdominal CT scans. Figure 1 provides an overview of the process. The input for the pancreatic detection process is CT scan images, which offer cross-sectional views of the pancreas and surrounding tissues. These images serve as the primary data source, as they provide the anatomical details necessary to identify and examine the pancreas. To improve image quality and standardize input dimensions, the collected images undergo a preprocessing process that includes resizing, cropping, and filtering.

1. **Resizing:** It involves uniformly resizing images to conserve computing resources and ensure compatibility with neural network design.
2. **Cropping:** This technique focuses on the region of interest (the pancreas and its surrounding areas) to enhance segmentation accuracy and eliminate extraneous background.
3. **Filtering:** It utilizes contrast enhancement and noise reduction techniques to enhance the visibility of tissue structures and organ boundaries, which is crucial for accurate segmentation.

Data augmentation is used after pre-processing to increase the model’s robustness and generalization. By artificially increasing the diversity of the training dataset, the model becomes more resilient to changes in patient anatomy, imaging circumstances, and scanner variations.

Then, a novel dual Self-attentive Transformer Unet (DSTUnet) model is proposed for accurate pancreatic segmentation. Our model incorporates dual self-attention Swin transformers on both the encoder and decoder sides to facilitate global context extraction and refine candidate regions. The fat fraction is estimated after the pancreas has been segmented using a DSTUnet. It is a measure of the percentage of fat in the pancreatic tissue. This study employs histogram analysis to automatically measure pancreatic fat, specifically the fat volume fraction (FVF).

### Data pre-processing

**Fig. 2:**
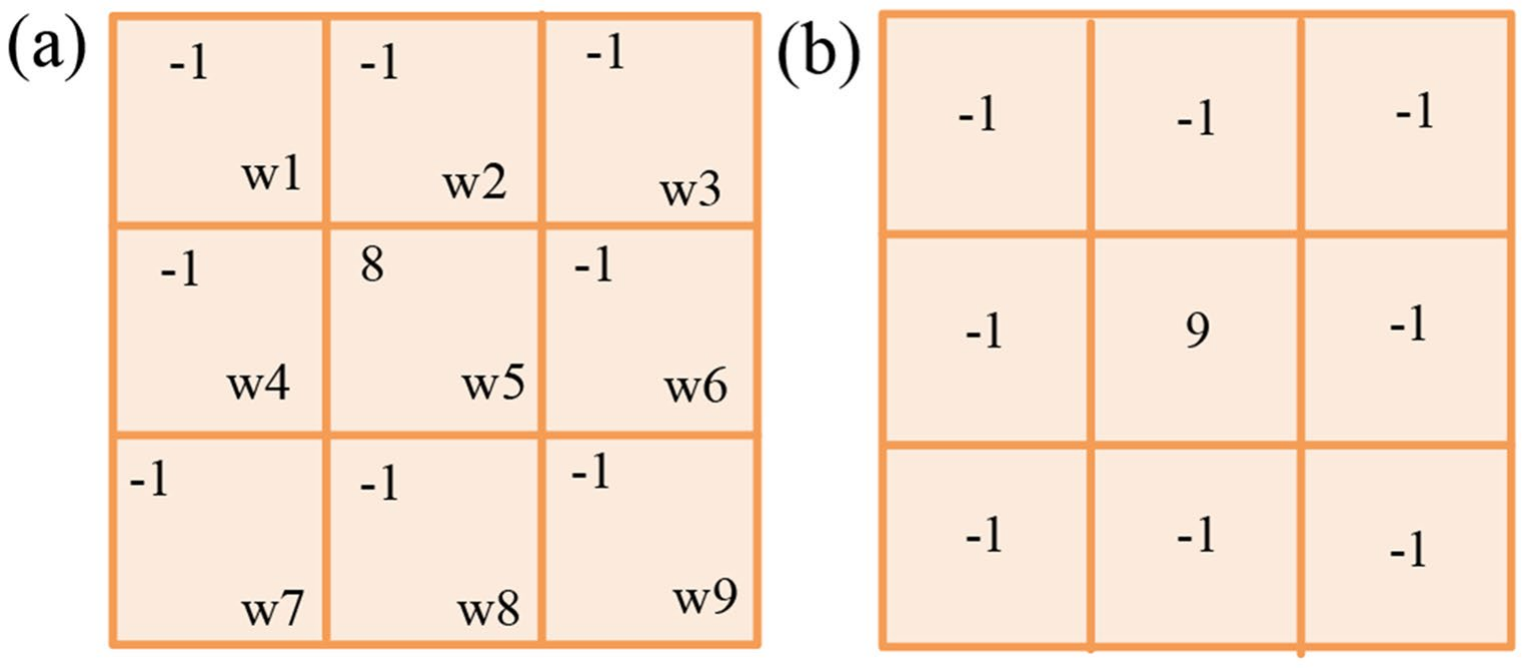
Filter (**a**) Laplacian filter (**b**) Sharpening filter

The image is interpolated utilizing nearest-neighbor interpolation in the depth direction and cubic spline interpolation in the transverse plane to resample all images to the uniform target resolution. After determining the Hounsfield unit (HU) range corresponding to the image labels, clipping is carried out between 0.55% and 99.4%. The images are then standardized. Throughout the inference procedure, sliding inference is performed using a window the same size as the patch, with a Stride length of Semi-patch size. Then, a Weighted Gaussian filtering technique is applied to mitigate the effects of irregularities near the image boundary during the fusion process. The images are first sampled, then sharpened using a filter before being fed into the segmentation model. The idea of this filter is derived from Laplacian filters, which show regions of abrupt intensity changes and are an example of second-order derivative system enhancement. Traditionally, this can be obtained using an Eq. (1) [31].

1 $${\nabla ^2}g = {{{\partial ^2}g\left( {s,t} \right)} \over {\partial {s^2}}} + {{{\partial ^2}g\left( {s,t} \right)} \over {\partial {t^2}}}$$

where $\frac{{{\partial ^2}g\left( {s,t} \right)}}{{\partial {s^2}}} = g\left( {s + 1,t} \right) + g\left( {s - 1,t} \right) - 2g\left( {s,t} \right)$ and $\frac{{{\partial ^2}g\left( {s,t} \right)}}{{\partial {t^2}}} = g\left( {s,t + 1} \right) + g\left( {s,t - 1} \right) - 2g\left( {s,t} \right)$. Here,$\,g\left( {s,t} \right)$ represents discrete 2D function used for representing the pixel intensity at coordinates $\left( {s,t} \right)$ in an image. ${\nabla ^2}g$ represents the sum of second partial derivatives in the$\,s\,$and $t$ directions after applying Laplacian operator to the function $g$. The second order partial derivative of $g$with respect to $s$ is represented as $\frac{{{\partial ^2}g\left( {s,t} \right)}}{{\partial {s^2}}}$, while the second order partial derivative of $g$with respect to$\,t$ is represented as $\frac{{{\partial ^2}g\left( {s,t} \right)}}{{\partial {t^2}}}$.

2 $${\nabla ^2}g = \left[ \matrix{ g\left( {s + 1,t} \right) + g\left( {s - 1,t} \right) \hfill \cr + g\left( {s,t + 1} \right) + g\left( {s,t - 1} \right) \hfill \cr} \right] - 4g\left( {s,t} \right)$$

where, $g\left( {s \pm 1,t} \right)$ and $g\left( {s,t \pm 1} \right)$ represent the horizontal and vertical pixel intensities at the closest neighbors of $\left( {s,t} \right)$. One can create a mask using Eq. (2). One of the variations of the Laplacian filter is applied in this investigation. Figure 2(a) displays the filter employed in this investigation.

The intensity value obtained from the provided Laplacian filter is calculated as the sum of the mask’s left point and the remaining points, which are calculated as follows: ${\omega _5} + \left( {{\omega _{1 + }}{\omega _2} + {\omega _3} + {\omega _4} + {\omega _6} + {\omega _7} + {\omega _8} + {\omega _9}} \right)$. Here, the left value and the other related values are added to determine the intensity value “0”. Once more, filtering an original image through this mask yields a dark image with only the edge of the image detected at the 0-intensity value. The following guidelines in (3) can be used to create the original image [32].

3 $$h\left( {s,t} \right) = \left\{ {\begin{array}{*{20}{c}} {g\left( {s,t} \right) - {\nabla ^2}g\,\,\,;\,\,{\omega _5} < 0\,} \\ {g\left( {s,t} \right) + {\nabla ^2}g\,\,\,;\,\,{\omega _5} < 0} \end{array}} \right.$$

where, $h\left( {s,t} \right)$ represents the final pixel value at the location $\left( {s,t} \right)$ after enhancing the image. ${\omega _{1,\,}}{\omega _2}, \ldots {\omega _9}$ are the pixel intensity values in a $3 \times 3$ neighborhood centred at ${\omega _5} = g\left( {s,t} \right)$. Therefore, the first condition of (3) is satisfied when the middle value of the Laplacian filter is smaller than 0; Otherwise, the second condition is fulfilled. Consider $g\left( {s,t} \right) \to {\omega _5}$ and ${\omega _1},{\omega _2}, \ldots {\omega _9}$ for the remaining position. Equation (4) can be expressed by considering the respective Laplacian mask from Eq. (3).

4 $$\eqalign{ h\left( {s,t} \right) = & 9{\omega _5} - {\omega _1} - {\omega _2} - {\omega _3} \cr & - {\omega _4} - {\omega _6} - {\omega _7} - {\omega _8} - {\omega _9} \cr} $$

Using Eq. (4), the produced mask is shown in Fig. 2(b). This sharpening filter is used to highlight and sharpen the Contours of images, and in addition, creates a transition between features that are more noticeable and recognizable than in flat, noisy, and blurred images. Overall, the Laplacian filter in Fig. 2(a) is a 3 × 3 kernel that emphasizes areas of abrupt intensity change for edge recognition and image enhancement. An 8 in the middle is surrounded by − 1 in the kernel values. The identity matrix and the Laplacian operator are combined to form the contrast-sharpening filter as shown in Fig.  2(b). By strengthening high-frequency components (edges, transitions), this arrangement sharpens the image by increasing the contrast between a pixel and its surroundings.

### Data augmentation

Practitioners can significantly increase the variety of the images for the neural networks by using data augmentation rather than collecting new data. Techniques for image augmentation could aid in addressing issues with data overfitting, enhancing training facilities, and lowering network generalization errors. This article created various images based on rescaling, zooming, horizontal flipping, and shearing processes using augmentation techniques on image data. These procedures were implemented to enhance the data.

### DSTUnet for pancreas segmentation

**Fig. 3:**
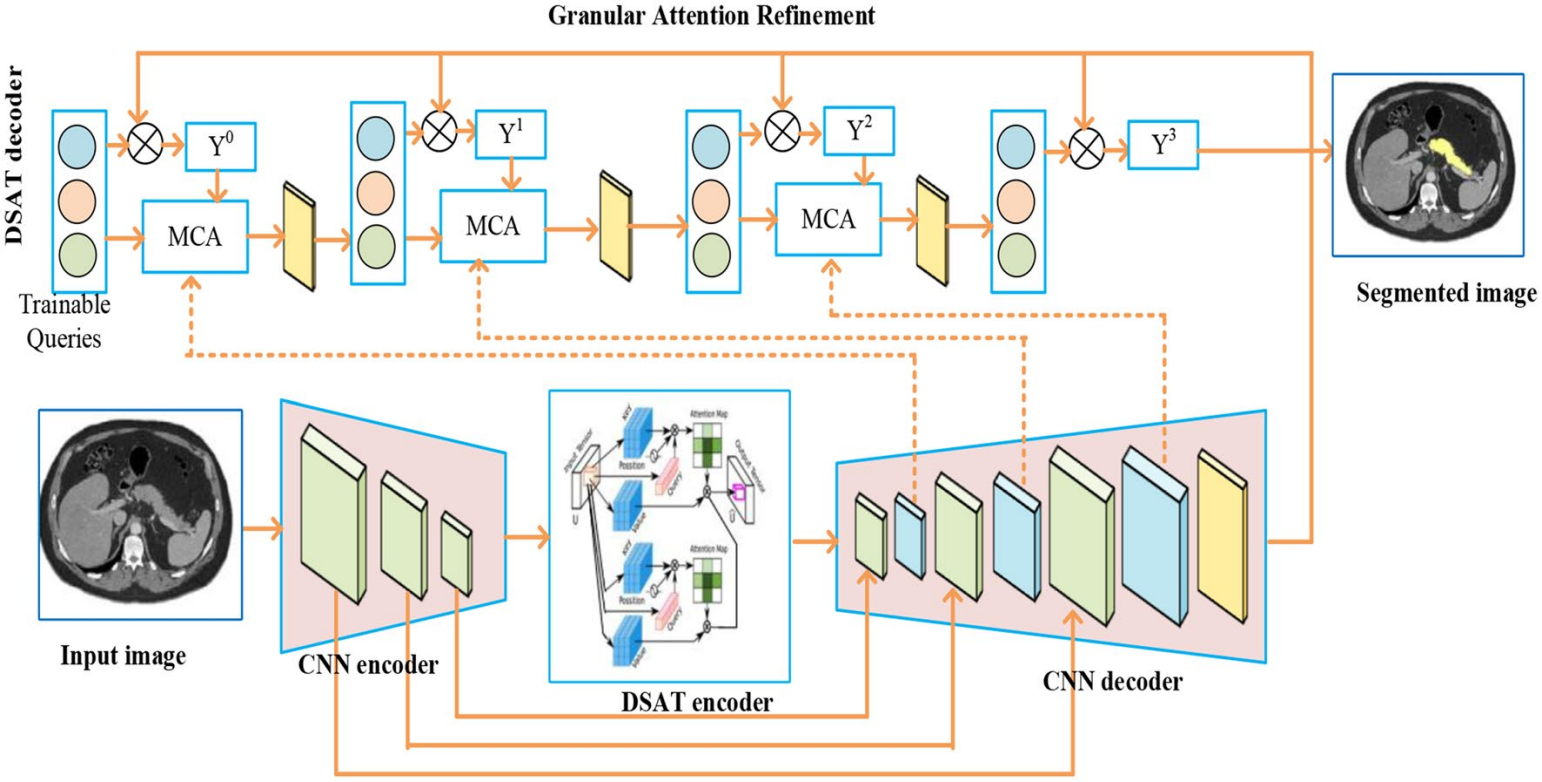
DSTUnet for pancreas segmentation

This study proposes a new DSTUnet model for automatically detecting the pancreas in abdominal CT scans. The traditional U-net model decodes the images to their full spatial resolution after encoding their high-level feature representations. The proposed model utilizes Dual Self-Attentive Transformers (DSAT) in both the encoding and decoding stages of traditional U-net systems to explore detailed attention strategies. Figure 3 depicts the structure of the DSTUnet model.

The CNN encoder, CNN decoder, DSAT encoder, and DSAT decoder are the four parts of DSTUnet. A CNN is initially utilized as a feature-extracting unit to create a feature map for the input. DSAT is thus used as both an encoder and a decoder. CNN. CNN extracts low-level features, such as edges, textures, and shapes, which are essential for defining the boundaries of the pancreas. The primary innovation of this framework is DSAT. Its components include Swin transformers [33], which are hierarchical view transformers with transform windows of various sizes used to record global context information and long-range dependencies. To segment small, ambiguous organs like the pancreas, the dual attention mechanism enhances spatial feature refinement, capturing both intra-region similarity and inter-region connectivity. CNN decoders use convolutions and up-sampling layers to replicate the encoder and restore the original image’s spatial resolution. Multi-head cross-attention (MCA) modules and trainable queries form the DSAT decoder. The MCA blocks enable the model to concentrate on pertinent regions by matching queries with feature maps. By combining contextual and spatial clues, each step of granular attentional refinement improves the predictions provided by the previous one. Compared to utilizing a conventional Transformer as the encoding unit, the hybrid CNN-DSAT encoding unit performs better.

#### DSAT encoder

**Fig. 4:**
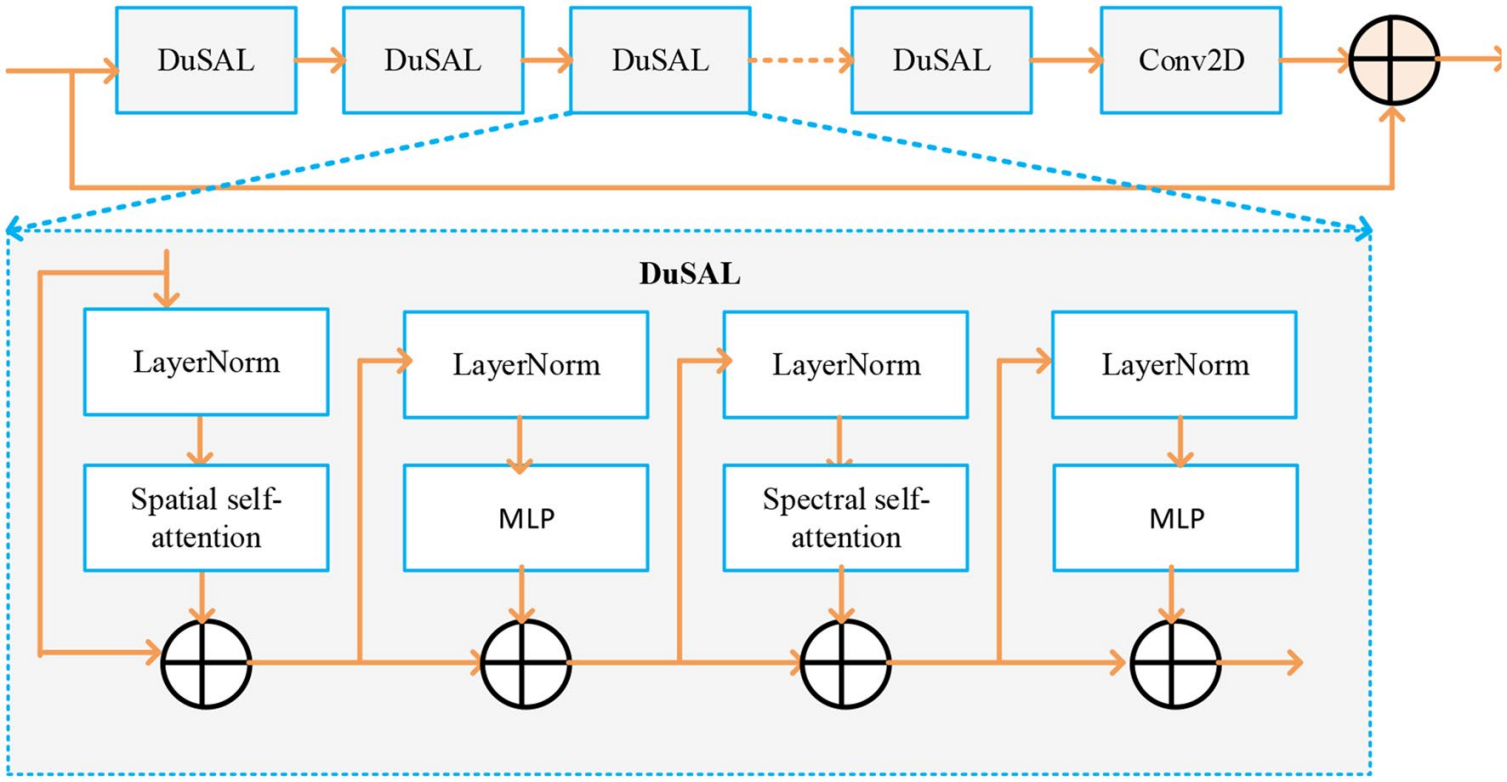
DSAT encoder

The internal structure and operational flow of the DSAT (dual self-attenuating transformer) encoder, a key component of the proposed DSTUnet design, are depicted in Fig. 4. Four DuSAL (dual self-attention layers) form its stack, followed by a Conv2D operation and element-wise addition for the final residual connection. Layer Normalization (LayerNorm), Spatial Self-Attention Module, Spectral Self-Attention Module, Multi-Layer Perceptrons (MLPs), and Residual Connections are the primary modules that comprise each DuSAL (Dual Self-Attention Layer). LNorm is used to stabilize and speed up training before every attention and MLP block. The Spatial Self-Attention Module captures dependencies and relationships between various spatial locations in the image. By focusing on inter-channel interactions, the Spectral Self-Attention Module enables the model to recognize and emphasize feature combinations most relevant to organ-level semantics.

As illustrated in Fig. 4, the DSAT module includes a 2-D convolution block and $N$ dual self-attention layers (DuSALs). It is employed to extract intermediate features iteratively ${f_{p,q}}$from CNN features ${f_{CNN}}$ as in (5)

5 $${f_{p,q}} = {\mathcal{M}_{DuSAL}}\left( {{f_{l,q - 1}}} \right),\,\,\,\,\,\,\,\,\,\,\,\,\,\,\,\,q = 1,2, \ldots N$$

where$\,{f_{p,q}}$ indicates the intermediary feature map at the $q - th$ DuSAL within $p - th$ DSAT.

${\mathcal{M}_{DuSAL}}$ represents the $q - th$ DuSAL unit is made up of the MLP block, layer norm, spectral self-attention, and spatial self-attention. The network then uses residual connections to deliver the DSAT output ${f_{p,out}}$ as in (6)

6 $${f_{p,out}} = {\mathcal{M}_{conv}}\left( {{f_{p,N}}} \right) + {f_{p,0}}$$

Where is the final output of DSAT following convolution and residual connection ${\mathcal{M}_{conv}}$ is a 2D convolution operation applied after DuSAL and it is used for refining and combining features. The total number of DuSAL layers in each DSAT block is denoted by $N$.

The DuSAL is used to reduce distortion, improve the performance of nonlocal long-range dependencies, and extract deep features from images. The Swin transformer serves as the foundation for the DuSAL. The primary variance between DuSALs and Swin transformers is the use of self-attention.

There are two steps in the DuSAL. Initially, it uses the Swin transformer’s Swin attention to learn spatial information. The DuSAL component utilizes a Swin transformer to acquire spatial knowledge. This integration is performed in the spatial self-attention step. Here, the main function of Swin attention is to facilitate effective spatial self-attention within local, non-overlapping windows. The shallow feature ${f_{CNN}}$is initially divided into ($\tilde h \times \tilde w/{n^2}$) nonoverlapping local windows ($\tilde h$: height, $\tilde w$: width, $n$: window size) during spatial self-attention. A Swin mechanism is then used to calculate self-attention for each window. This implies that the standard self-attention operation is applied within the pixels of each local window, rather than being applied across the feature map. This is the specialty of Swin Transformers. The query, key, and value for the local window $\tilde S$ are computed as in (7)

7 $$\tilde Q = \tilde S{N_Q},\,\tilde K = \tilde S{N_K},\,\tilde V = \tilde S{N_V}$$

Where $\tilde h$and $\tilde w$ stand for the input feature map’s height and width. $\tilde S$ is the non-overlapping window of size $n \times n$ that was taken from the shallow feature map ${f_{CNN}}$. The size of the window utilized for swin attention is $n$. Each window’s query, key, and value matrices are denoted by $\tilde Q$, $\tilde K$, $\tilde V$. The ${N_Q}$,$\,{N_K}$, and ${N_V}$ are the forecast matrices for $\tilde Q$, $\tilde K$, $\tilde V$. The attention in the window can be calculated using $\tilde Q$, $\tilde K$, $\tilde V$ matrices.

8 $$Attention\left( {\tilde Q,\tilde K,\tilde V} \right) = softmax\left( {\tilde Q{{\tilde K}^T}\sqrt d + P} \right)\tilde V$$

where $d$ stands for the dimensionality of the query/key vectors. $P$ is the learnable relative positional bias, which is utilized for preserving spatial information. The DSAL does not employ a global self-attention for spatial features. Instead, it uses the computationally efficient local attention mechanism of the Swin model. The output of this spatial attention (from different windows) is either merged or subjected to additional processing before being input into the spectral self-attenuation mechanism (the second stage of DSAL) and the next MLP layer.

Secondly, a spectral self-attention mechanism is integrated to investigate the deep frequency-domain information of CT images. It converts the input CT image of size $\tilde h \times \tilde w \times 1$ into a frequency domain utilizing the Fourier Transform (FFT) or Wavelet Transform (DWT) to get a feature representation of size$\tilde h\tilde w \times \tilde C$. The following eq. (9) yields the linear projections along the frequency dimension $(\overline {Q,} \bar K,\bar V$).

9 $$\bar Q = \tilde S{W_Q},\,\bar K = \tilde S{W_K},\,\bar V = \tilde S{W_V}$$

where the projection matrices across the spectral channel are denoted by ${W_Q}$, ${W_K}$, and ${W_V}$respectively. In the frequency domain, the self-attention mechanism is carried out using (10)

10 $$Attention\left( {\bar Q,\bar K,\bar V} \right) = softmax\,\left( {\sigma {{\bar K}^T}\bar Q} \right)\bar V$$

where $Attention\left( {\bar Q,\bar K,\bar V} \right)$ represents the output feature from multi-head self-attention and the matrix multiplication $\bar K$, $\bar Q$is reweighted by the parameter $\sigma$. The DSAT network uses a multilayer perceptron (MLP) layer to link spectral and spatial attention. The MLP consists of one ReLU nonlinear layer and two fully connected layers. It can enhance feature extraction by utilizing spectral and spatial self-attention techniques.

#### DSAT decoder

Medical image segmentation is typically viewed as a per-pixel classification task by conventional methods, such as U-Net. The per-pixel cross-entropy loss is generally used for training a segmentation model to categorize each pixel into one of the possible C categories. In this study, pancreas image segmentation is considered a mask classification problem rather than a pixel-by-pixel analysis. The idea of “organ query” is described wherein ${D_{dec}}$dimensional feature vector represents each organ in the image. The proposed model aims to separate an image into $C$ segmentation classes into $X$ different candidate regions using a specified set of $X$ organ searches. For ease of understanding, Symbol Definitions and Notations used for the DSAT decoder are defined as follows:

- C indicates the total number of semantic categories (classes) for segmentation. This would normally be C = 2 in the context of pancreas segmentation: one class for “pancreas” and one for “background”.
- $\tilde h \times \tilde w$ indicates the feature map height and width utilized in the decoder ${f_{p,out}}$. These are usually downsampled versions of the original image sizes.
- ${D_{dec}}\,i$s the dimension of the feature vector used by the decoder to represent each element query.
- ${D_{dec}}\,i$s the dimension of the feature vector used by the decoder to represent each element query.
- ${A^0} \in {\mathbb{R}^{\tilde h \times \tilde w \times {D_{dec}}}}$ indicates the initial Organ Queries for Pancreas Segmentation.
- ${D_{dec}}$-dimensional query vector that is specifically designed to find and query for pancreas-like features is stored at each position ($\tilde h \times \tilde w$) in ${Y^0}$.
- ${Y^0}$ indicates the Coarse Predicted Pancreas Segmentation Map. This is the initial prediction map at feature map resolution, pixel by pixel. After applying $sigT$, its values are usually 0 or 1, representing an initial binary guess as to whether each pixel at that coarse resolution is part of the background (0) or part of the pancreas (1). The output is transformed into a definitive binary prediction for the coarse map by $sigT\left( \cdot \right)$.

The dot product between the early organ queries ${A^0} \in {\mathbb{R}^{\tilde h \times \tilde w \times {D_{dec}}}}$and the final block feature of U-net ${f_{p,out}}$can be used to calculate the coarse predicted segmentation map as in (11)

11 $${Y^0} = sigT\left( {{A^0} \times f_{p,out}^T} \right)$$

where a Sharp cutoff filtering with a threshold of 0.6 is performed after sigmoid activation. DSAT decoder, which improves the coarse prediction ${Y^0}$ by refining organ queries. The attention method in every layer will enable the DSAT decoding unit to fully interact with image characteristics and capture inter-organ dependencies, similar to the DSAT encoder. Combining mutual attention with spatially adaptive multiple-scale CNN attributes improves anatomical structure queries in every decoding layer. It acknowledges the wealthy positioning in intermediary CNN attributes and enhances the Transformer’s global feature embedding.

#### Granular Attention Refinement

The DSAT decoder then proceeds to an iterative refinement phase utilizing Masked Cross-Attention (MCA) modules. This is the core of the “Granular Attention Refinement” block. This section highlights the importance of granular attention refinement, particularly for smaller target segmentations. This method uses a rough mask from the first step to direct further improvements. A mask attention module is integrated into the DSAT decoder to facilitate a smooth granular refinement process. To minimize ambient interference and emphasize the area of interest, this improvement aims to establish mutual attention within the foreground zone based on the previous coarse forecast for each category. This enhanced attention map supports later, precise segmentation phases iteratively. Here, the iterative refining process is initiated by considering the coarse-stage mask prediction as ${A^0}$and ${Y^0}$ respectively.

A key component of the DSAT decoder is the MCA (Masked Cross-Attention) module, which was specifically designed to utilize attention-guided algorithms to combine and modify learned representations. Here, attention is directed toward pertinent spatial or anatomical locations using a set of trainable query vectors. The mask regulates the scope of attention and directs it toward significant structures by suppressing unimportant areas. During attention computation, masked attention employs a masking technique to exclude or reduce the weight of parts of the input features that are deemed insignificant. The MHSA module extracts multi-scale relationships by letting each attention head concentrate on distinct aspects of the input characteristics.

**Fig. 5:**
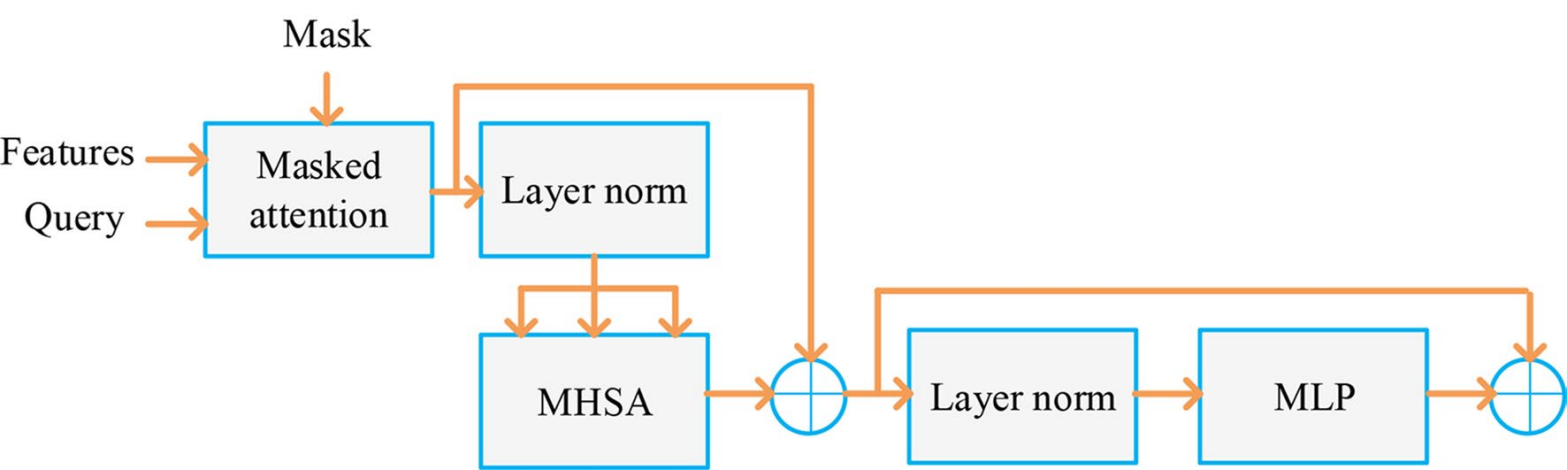
MCA structure

The current refined organ queries ${A^i}$, current coarse prediction mask ${Y^i}$and multiscale CNN features ${f_{CNN}}$ will be fed into MCA for every iteration $i$. The attention calculation is carried out by the MCA module as seen in Fig. 5:

12 $$\eqalign{& {A^{i + 1}} \cr & = {A^i} + softmax\left( {\left( {{A^i}{W_Q}} \right){{\left( {f_{CNN}^i{W_K}} \right)}^T} + l\left( {{Y^i}} \right)} \right) \cr & \,\,\,\,\,\, \times {f_{CNN}}{W_V} \cr} $$

13 $$l\left( {{Y^i}} \right) = \left\{ {\begin{array}{*{20}{c}} {0\,\,\,\,\,\,\,\,\,if\,{Y^i} = 1} \\ { - \infty \,\,\,\,\,\,\,\,\,\,otherwise} \end{array}} \right.$$

where the weight matrix ${W_Q}$ is used to create queries for the following layer by linearly projecting the$\,i$th query characteristics. This technique enables the queries to attend to pertinent image features (${f_{CNN}}$) by suppressing attention to background regions based on the mask ${Y^i}$. The parametric weight matrices ${W_K}$ and ${W_V}$ are also used to convert the U-Net feature ${f_{CNN}}$ into keys and values. The softmax function ensures that attention weights are normalized. The proposed transformer decoding unit analytically improves the segmentation outputs over several rounds through the iterative update of the Anatomical structure queries and the accompanying mask forecasts.

Following the last iteration, the dot product with the final block features of U-Net ${f_{CNN\,}}$may decode the updated organ query ${A^T}$ revert to the final enhanced binary segmentation map ${Y^T}$. Additionally, a linearized layer is employed with a weight ${W_{fc}}$ that endeavors the sophisticated organ embedding ${A^T}$ to the output category logits $Out$ in order to assign each binarized mask to a single semantic class using (14).

14 $$\,\,\,\,\,\,\,\,\,\,\,\,\,\,\,\,\,\,\,\,\,\,\,\,\,\,\,\,\,Out = {A^T}{W_{fc}}$$

This formula demonstrates the transformation of a highly optimized, high-dimensional grid query (${A^T}$) into a low-dimensional output suitable for direct classification (e.g., signaling the presence or confidence of the pancreas mask). The proposed model aligns forecasts and ground-truth segments using the Hungarian matching loss and integrates a Transformer-based encoder and decoder into the U-Net. For every segmented prediction, it combines binary mask loss and pixel-wise classification loss as in (15):

15 $$L = \alpha \left( {{L_{ce}} + {L_{dice}}} \right) + \beta {L_{clas}}$$

where the first term shows the per-pixel segmentation loss $\alpha \left( {{L_{ce}} + {L_{dice}}} \right)$. It combines binary cross-entropy loss ${L_{ce}}$ and dice loss ${L_{dice}}$. The second term denotes the mask classification loss. For every candidate region, the cross-entropy loss instantiates the categorization loss ${L_{clas}}$. The hyper-parameters$\,\alpha$ and $\beta$ are utilized to balance the mask classification loss and the per-pixel segmentation loss.

### Fat fraction estimation

The relationship between metabolic disorders and ectopic fat deposition in the pancreas is becoming increasingly significant. CT images can assess the proportion of pancreatic fat. Many diseases have been linked to changes in pancreatic volume. To alleviate the burden of these diseases, it is anticipated that measuring disparities in pancreatic volume will provide new insights for diagnosis and treatment. The CT Hounsfield unit of − 20 HU is used to characterize fat deposition in the pancreas while estimating pancreatic volume [30]. However, considering that − 10 HU could overstate the fatty tissue volume due to the influence of fractional volume. Also, − 30 HU could understate the Fatty tissue volume. For unclear reasons, fat deposits preferentially collect within the pancreas interstitial septa and tend to protect Langerhans acini and islets (intralobular spaces). Thus, a rise in interlobular fat can be detected at a threshold of − 20 HU. The complete workflow of the proposed method is provided in Algorithm 1.

**Algorithm 1:**
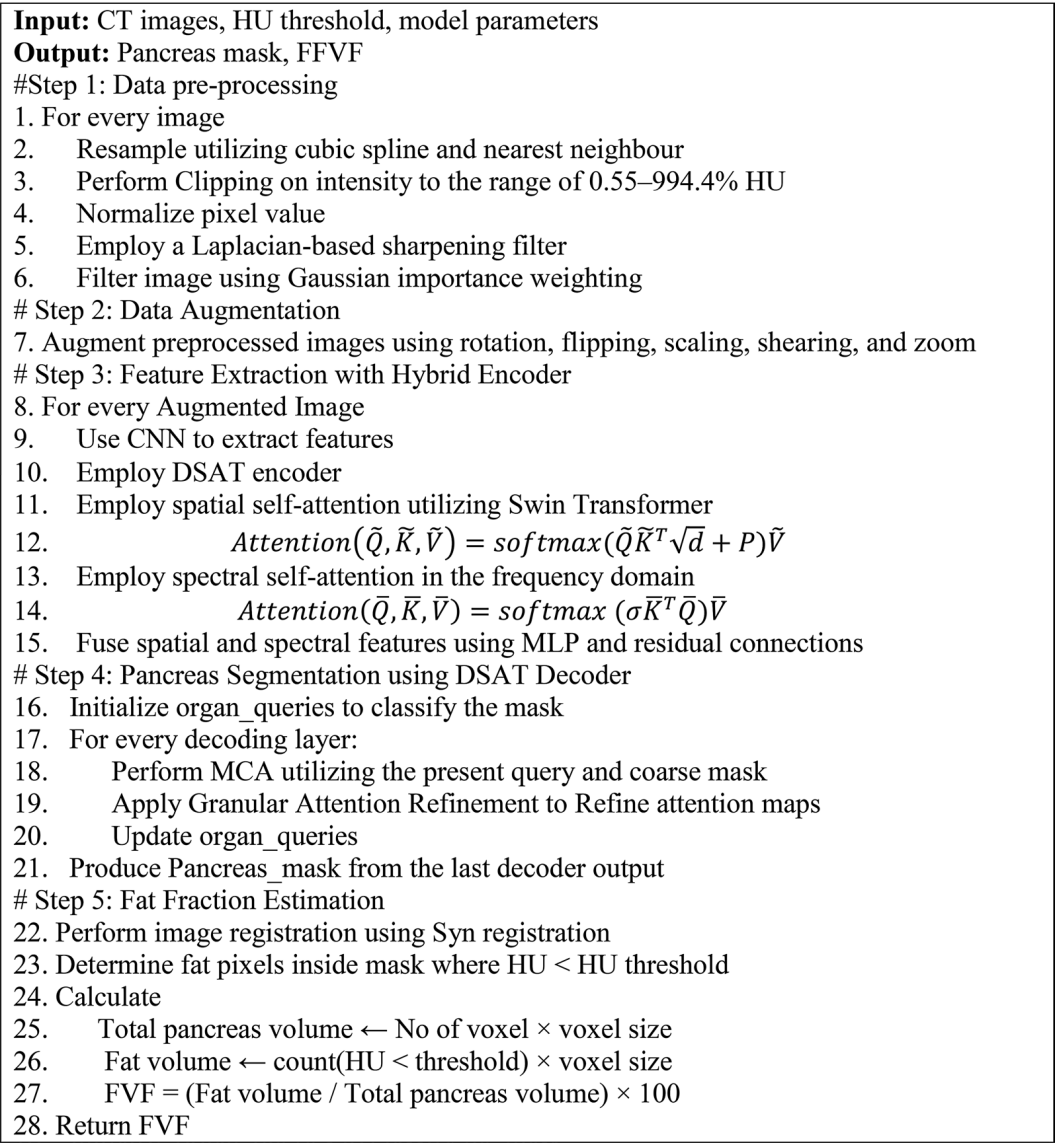
Proposed pancreatic segmentation and fat fraction estimation

## Results and discussion

Simulating the suggested DSTUnet using Python and training it on an NVIDIA RTX8000 processor confirms its performance. We test our approach on five distinct datasets: The AMOS dataset [34] contains 200 scans with segmentation masks of multiple organs, including the pancreas; WORD [35] includes 120 pancreas segmentations of healthy persons; BTCV [36] provides 30 pancreas segmentations of healthy persons; and ITK-SNAP [29] comprises 220 abdominal CT scans from 227 patients. The AMOS dataset consisted of 200 abdominal CT scans; 140 of these scans were randomly selected for training, 30 for validation, and the remaining 30 for testing. In the WORD dataset, eighty scans were used for training, twenty for validation, and twenty for testing. Due to the small sample size, all 30 images in the BTCV dataset with pancreatic annotations were used in the 5-fold cross-validation procedure. The ITK-SNAP dataset comprises 220 annotated abdominal CT scans, with 154 scans used for training, 33 for validation, and 33 for testing.

Here, identical experimental conditions are considered for DSTUnet and existing methods to ensure a fair comparison across all currently used methods [37, 38]. Table 2 provides implementation details, including the suggested model’s architecture, hyperparameters, and training parameters.

**Table 2:** Simulation parameters

Parameters	Values
Batch size	2
Crop size	40×224×224
Learning rate	3${e^{ - 4}}$
Optimizer	Adam
Hungarian matching loss $\alpha$ and $\beta$	0.7 and 0.3

**Fig. 6:**
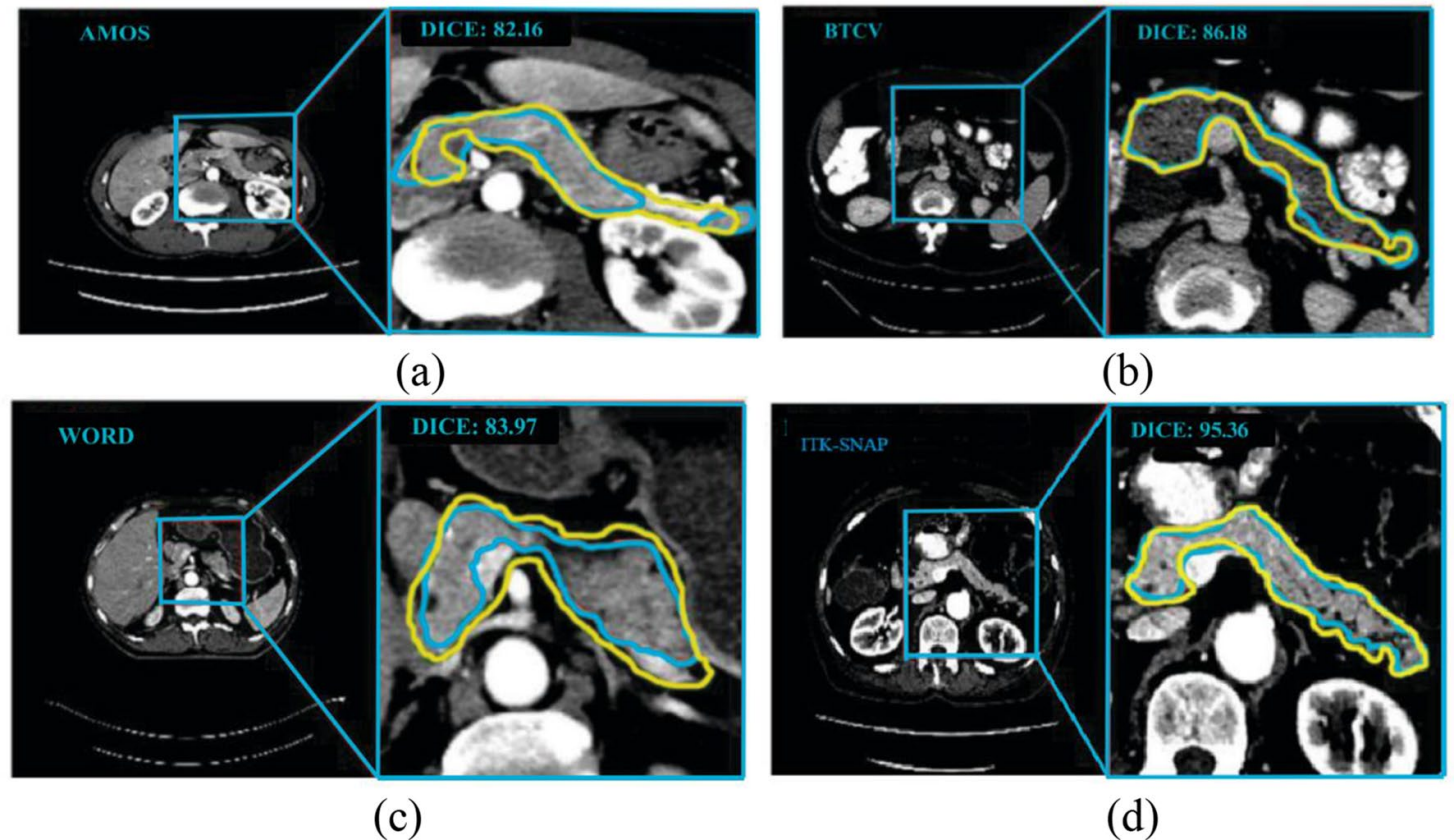
Visual examination of the segmentation results (**a**) AMOS dataset [34], (**b**) WORD dataset [35], (**c**) BTCV dataset [36], (**d**) ITK-SNAP dataset [29]

The visual analysis of the segmentation outcomes on various datasets is shown in Fig. 6. Each image displays a CT scan focusing on the pancreas region. The blue contour shows the segmentation of the ground truth. The yellow contour shows the model’s projected segmentation. The overlapping of the blue and yellow outlines indicates the degree to which the model matches the pancreatic border. The ITK-SNAP performs the best segmentation, as seen by its highest Dice score of 95.3. This is because it encompasses a wide range of CT scans from various patients, different scanner configurations, and diverse environmental conditions. This diversity reduces segmentation errors and enhances the model’s ability to generalize.

### Evaluation of the segmentation model

In Table 3, two complementary categories of measurements were used to thoroughly evaluate the segmentation effectiveness: boundary-based measurements, such as the 95% Hausdorff Distance (HD95) and Average Symmetric Surface Distance (ASSD), and region-level measurements, including the Dice score (Dice), Jaccard index (Jaccard), Precision, and Recall. The suggested model achieved an average dice score of 82.16% on the AMOS dataset, with an HD95 distance of 9.12 mm. Alternatively, the ITK-SNAP dataset achieved an average dice score of 95.36% with an HD95 distance of 2.32 mm. It demonstrates that the segmentation accuracy decreased when the proposed trained model was employed on the AMOS, WORD, and BTCV external datasets, in contrast to the ITK-SNAP dataset. The distribution of these datasets may differ from that of the ITK-SNAP dataset and may reflect actual clinical situations.

**Table 3:** Analysis of segmentation results on different datasets

Metrics	Dataset							
	***AMOS***		***WORD***		***BTCV***		***ITK-SNAP***	
	PanSegNet [24]	DSTUnet	PanSegNet [24]	DSTUnet	PanSegNet [24]	DSTUnet	nnTransfer [32]	DSTUnet (Proposed)
Dice (%)	78.79	82.16	80.89	83.97	83.71	86.18	93.7	95.36
Jaccard (%)	67.96	73.94	68.51	72.88	72.43	77.18	88.5	91.82
Precision (%)	80.39	84.37	85.47	88.91	81.84	85.19	93.7	95.74
Recall (%)	80.37	83.99	78.17	81.32	86.17	89.98	94.0	95.12
HD95 (mm)	13.47	9.12	12.85	9.72	8.29	6.94	2.7	2.32
ASSD (mm)	2.92	1.29	2.75	1.76	1.59	1.17	-	0.96

Here, PanSegNet [24] performs poorly compared to the suggested DSTUnet. Its main feature is the linear self-focusing capability of CNNs. CNNs are less successful at collecting long-range relationships in the pancreas due to their local receptive fields. The pancreas has uneven forms and hazy boundaries. nnTransfer [32] depends on cross-domain knowledge transfer. This model’s source and target domains are not isomorphic (have different structures or data distributions). However, information from other medical imaging modalities may not transfer well to pancreatic segmentation, as pancreatic segmentation from abdominal CT images is a highly domain-specific process. As a result, it performs worse than the suggested DSTUnet. Moreover, the effectiveness of the proposed model is confirmed in terms of training loss and dice coefficients.

Table 4 compares the proposed model with other relevant models, such as Swin-Unet [39], TransUNet [40], and lightweight ViT [41], for pancreas segmentation. The considerable morphological heterogeneity and uncertain boundaries of the pancreas present considerable challenges to existing medical image segmentation methods, including previous transformer integrations (TransUnet and Swin-Unet). Despite providing a global context, early transformer designs exhibit difficulties in direct global interactions when employing window-based techniques, such as Swin-Unet. By modeling pancreas segmentation as a mask classification task and using a unique DSAT decoder with granular attention refinement, the proposed DSTUnet overcomes these limitations. This iterative technique utilizes Masked Cross-Attention to focus on pertinent anatomical regions, guided by dynamically generated coarse masks. As a result, it reduces ambient interference and improves adaptive feature interaction for accurate boundary delineation. This novel approach consistently outperforms competitors like Swin-UNet (94.24% Dice, 3.21 mm HD95) and TransUNet (94.14% Dice, 2.97 mm HD95), indicating its significant advantages in achieving higher accuracy and more robust segmentation, as well as superior performance across various metrics and datasets.

**Table 4:** Comparative Analysis of transformer models

Metrics	Dataset	Swin-UNet	TransUNet	lightweight ViT	DSTUnet
Dice (%)	AMOS	80.92	81.56	80.10	82.16
	WORD	82.51	83.28	81.87	83.97
	BTCV	85.02	85.89	84.51	86.18
	1TK-SNAP	94.24	94.14	93.90	95.36
Jaccard (%)	AMOS	71.88	72.51	70.92	73.94
	WORD	71.54	71.85	70.42	72.88
	BTCV	75.84	76.85	74.95	77.18
	1TK-SNAP	88.72	89.64	87.64	91.82
HD95 (mm)	AMOS	10.85	10.12	11.63	9.12
	WORD	11.05	10.49	11.79	9.72
	BTCV	7.56	7.12	8.31	6.94
	1TK-SNAP	3.21	2.97	3.57	2.32
ASSD (mm)	AMOS	2.10	1.78	2.68	1.29
	WORD	2.42	2.25	2.86	1.76
	BTCV	1.75	1.35	2.16	1.17
	1TK-SNAP	1.62	1.24	1.98	0.96

**Fig. 7:**
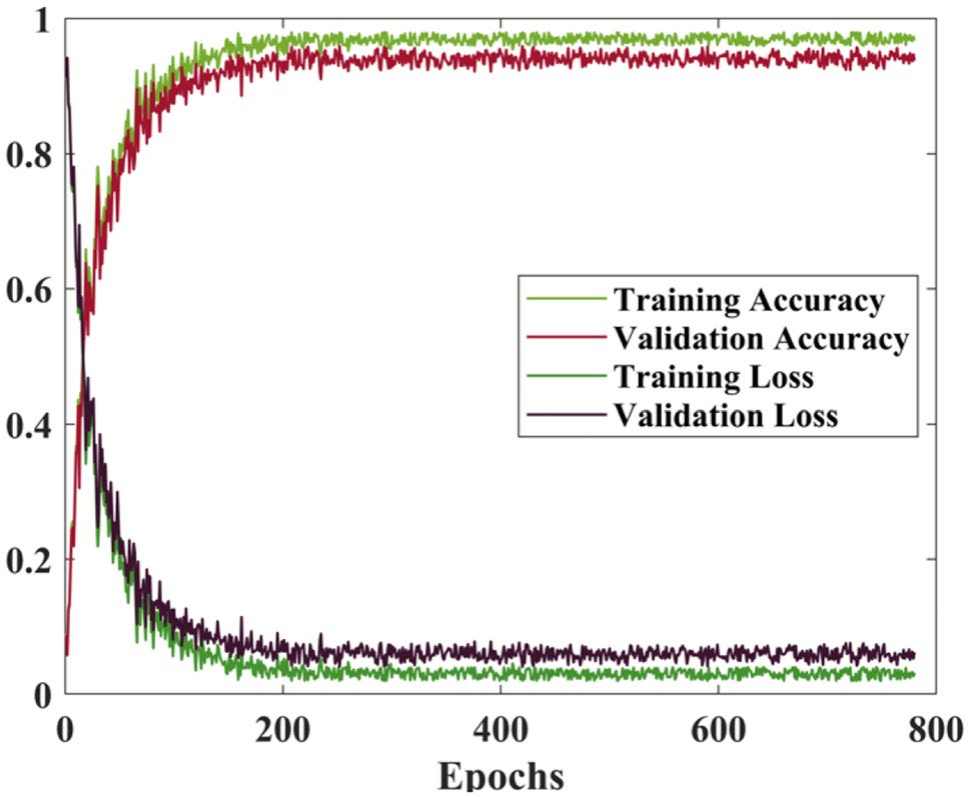
Plot of Dice coefficient accuracy and training loss

Figure 7 illustrates the training and validation performance of the suggested DSTUnet model across 800 epochs. According to Fig. 7, the suggested DSTUnet required fewer training iterations to reach a loss of 0.07, which it did at epoch 115 with a validation value of − 0.08. The training accuracy, which rises quickly and stabilizes at nearly 99%, indicates an intense match to the training data. The curves stabilize with minor variations after epoch 200, indicating that the model is neither over-fitting nor under-fitting and that the training process is smooth. The little difference between the training and validation curves indicates a balanced model with a well-regulated learning capability. This result also suggests that the proposed network was more adept at reaching the global optimum and converged more quickly.

### Evaluation of volume calculation and fat fraction estimation

**Fig. 8:**
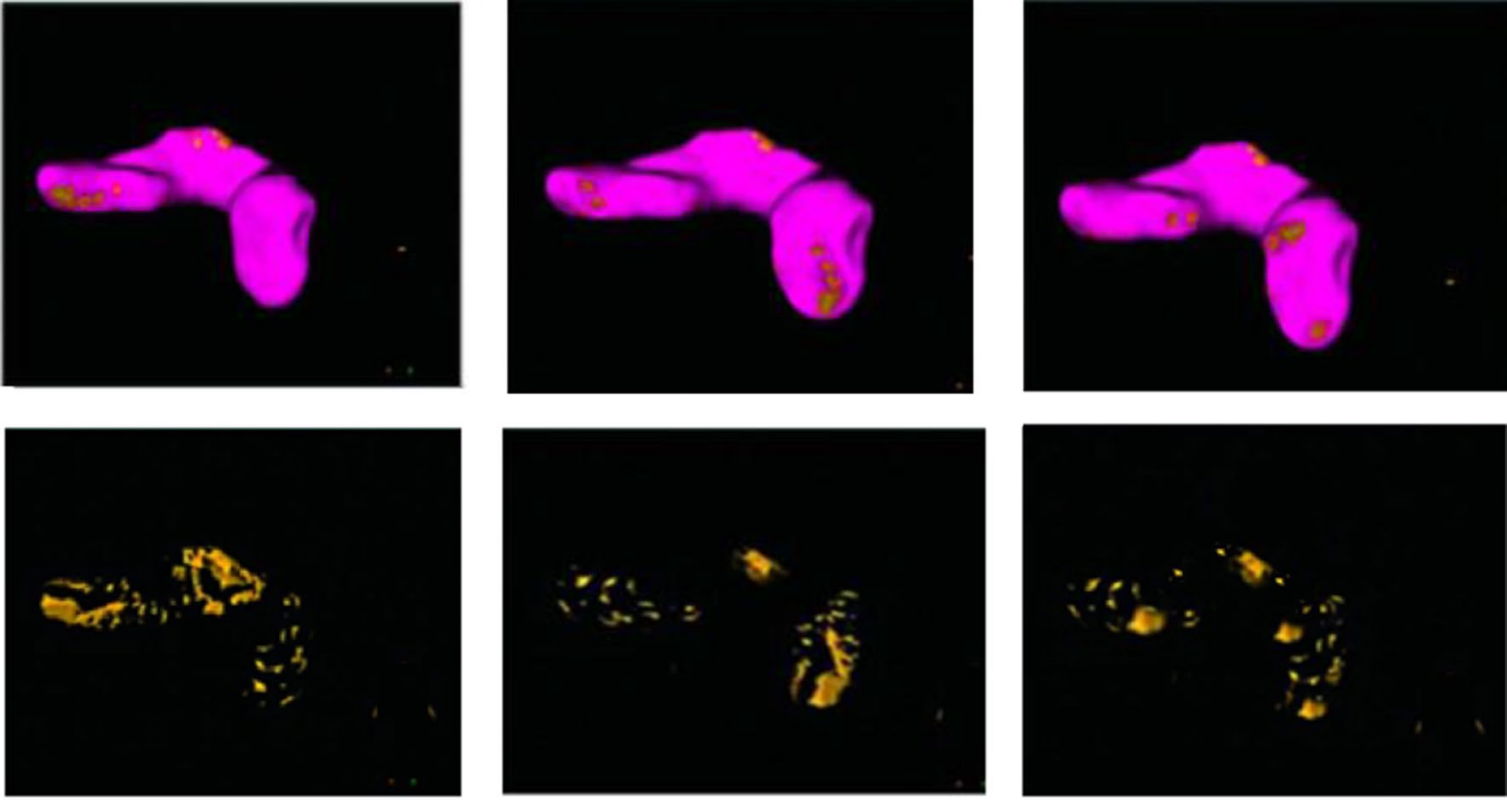
Visualization of fat infiltration

To determine the fat fraction, we segmented the pancreas using CT images and registered the segmented findings to the mask images. The Syn registration approach was selected as the best strategy after using Advanced Normalization Tools (ANTs) for registration. Hounsfield Units (HU) on CT images are commonly used to measure the amount of fat in the pancreas. Additionally, several procedures are involved in calculating pancreatic volume from segmented CT scans, including segmentation, calculation of voxel size, and volume computation. In DICOM information, voxel size is under Slice Thickness and Pixel Spacing. Following segmentation, the number of voxels identified as pancreas in each CT slice is counted to calculate the pancreatic volume. The following formula is then used to determine the segmented pancreas’ volume:

16 $$PV = M \times \left( {{U_{spacing}} \times {V_{spacing}} \times {W_{spacing}}} \right)$$

where $M$ stands for the total number of pancreatic voxels, ${U_{spacing}}\,$for voxel width in millimeters, ${V_{spacing}}$ for voxel height in millimeters, and ${W_{spacing}}$ for slice thickness in millimeters. The ensuing diagnostic findings of fat penetration in one instance are shown in Fig. 8. An unequal fat distribution in the Proximal, central, and distal pancreas is a common sign of pancreatic fat degradation. According to the visualization results, measuring the pancreatic CT fat score at a threshold of − 20 HU may be a feasible, non-invasive, and viable method for evaluating pancreatic fat degeneration. Figure 8 shows that the fat infiltration percentage was 8.9%, and the pancreatic volume was calculated to be 90.16 ml.

Table 5 displays the projected results on ITK-SNAP. The radiologists’ segmented pancreatic volume was regarded as the gold standard for training and assessing the segmentation model. 210 cases were randomly allocated to model training, and 19 cases to testing. The table confirms the accuracy of our model’s fat fraction estimation component by incorporating the ground truth values and error metrics. For example, the hypothetical average Fat Volume % Error of 0.84% and FVF % Error of 0.91% would suggest that our model’s fat estimating capabilities are highly accurate.

**Table 5:** Estimated fat volume outcomes on ITK-SNAP

Number of test images = 19	Pancreatic volume (ml) (estimated)	Fat volume (ml) (estimated)	Fat volume (ml) (Ground Truth)	Fat volume % error	FVF (%) (estimated)	FVF (%) (Ground truth)	FVF % error
Average	92.42	13.16	13.05	0.84%	13.37	13.25	0.91%
Range	21.67–127.86	2.71–32.97	2.60–33.10	0.5–2.0%	3.47–35.46	3.30–35.50	0.3–2.5%

A straightforward measurement for volume statistics is the relative volume prediction error (${E_v}$), which is written as follows:

17 $${E_v} = \frac{{{V_{predicted}} - {V_{real}}}}{{{V_{REAL}}}}$$

**Fig. 9:**
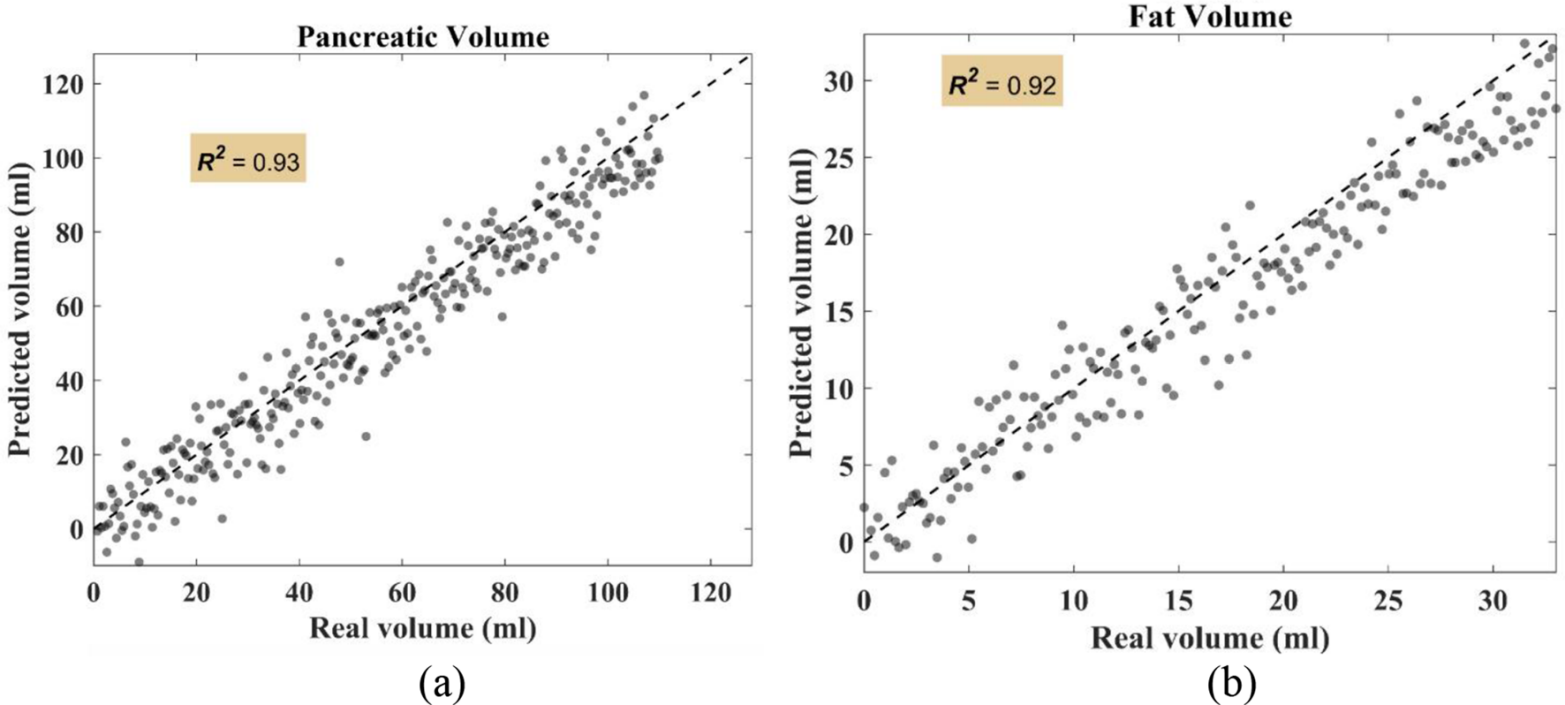
Correlation analysis (**a**) Pancreatic volume prediction (**b**) Fat volume prediction

Figure 9 displays a strong correlation between the actual pancreatic and fat volumes and the volumes anticipated for pancreas segmentation. It shows a linear fitting line with respective$\,{R^2}$ values of 0.93 and 0.92 for pancreatic volume and fat volume, respectively. These excellent ${R^2}$values support the potential usefulness of our volume prediction algorithm in clinical settings by illuminating its efficacy and accuracy.

**Fig. 10:**
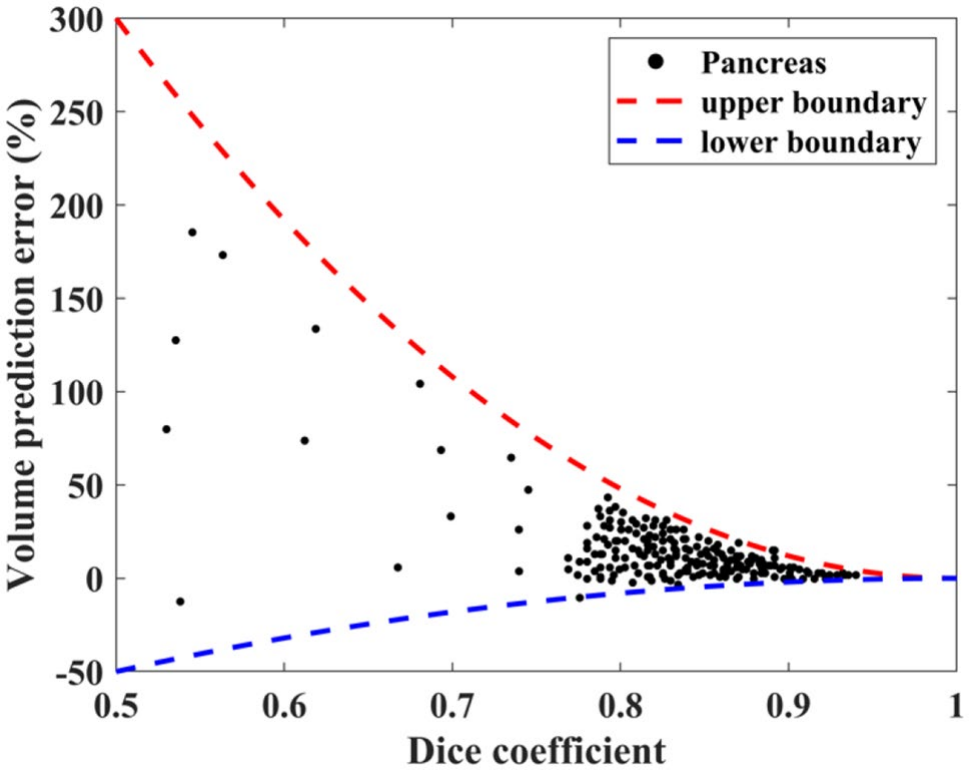
Volume error analysis

Figure 10 depicts the association between volume error and dice coefficient. Here, the error is illustrated using red and blue curves. These curves represent the maximum and minimum bounds in volume errors, showing the medically beneficial Dice scores. A maximum volume error of 20% is frequently considered reasonable for demographic and diagnostic research, suggesting that a dice score above 92% is appropriate in hospitals. The proposed method yielded satisfactory results with errors of 2% and 4%. This guarantees precise intraoperative guiding and preoperative evaluation.

The suggested model’s inference efficiency is then assessed by comparing it with several Unet versions, including nnUNet [34], nnFormer [35], PanSegNet [22], and nntransfer [30], as shown in Table 6. The same profiling scripts were used for all measurements on an NVIDIA RTX8000 CPU. Here, the same crucial hyperparameters were used to guarantee a fair comparison. Due to dual self-attention, the proposed model added minimal computational burden compared to the conventional model, nnUNet [34]. However, it provides much better accuracy than traditional designs; hence, this complexity is negligible.

**Table 6:** Model complexity and inference analysis

Model	Gflops (G)	Inference time (s)	Parameter (M)
nnUNet [34]	549.5976	0.1114	30.97
PanSegNet [22]	552.3234	0.1152	43.92
nnFormer [35]	696.1242	0.1241	49.25
nntransfer [28]	612.1728	0.1193	47.36
DSTUnet	558.1419	0.1159	44.06

### Ablation study

Ablation studies are crucial for evaluating the effectiveness of various deep learning model components in collaboration. In this study, the ablation studies compare the DSAT-UNet model with U-Net (without attention), U-Net + CNN Encoder, U-Net + Single Self-Attention, and U-Net + Transformer Encoder Only. This makes it easy to determine whether dual self-attention improves segmentation. The effectiveness of each network in the suggested DSAT-UNet model on the AMOS dataset is compared in Table 6. A 77.61% Dice score, 69.10% Jacquard code, and relatively high HD95 and ASSD values (11.94 mm and 3.01 mm, respectively) are all achieved using the basic U-Net model. All metrics improve slightly when using a CNN-based encoder instead of the default encoder; the Dice score improves to 79.82% and the HD95 improves to 11.42 mm, indicating improved feature extraction. When a single self-attentive module is added, the DICE increases to 80.32% and the ASSD decreases to 2.41 mm. This demonstrates how attentional processes enable the model to focus on the appropriate spatial regions, thereby improving delineation. The Standard U-Net performs the worst because it lacks transformer-based upgrades and self-attention mechanisms. On the other hand, Transformers enhance segmentation by improving the learning of long-range feature dependencies. Dual Self-Attention enhances segmentation by improving spatial and feature-wise interdependence compared to all other approaches. The complete DSAT-UNet model outperforms all others, achieving a Dice of 82.16%, the highest precision (84.37%), and the lowest HD95 (9.12 mm) and ASSD (1.29 mm). This Table emphasizes the significance of attention processes in improving semantic segmentation in biological applications and provides compelling evidence in favor of the suggested architectural decisions.

**Table 7:** Ablation study

Models	Dice (%)	Jaccard (%)	Precision (%)	Recall (%)	HD 95 (mm)	ASSD (mm)
U-Net	77.61	69.10	79.67	78.91	11.94	3.01
U-Net + CNN Encoder	79.82	70.14	80.17	79.96	11.42	2.85
U-Net + Single Self-Attention	80.32	71.53	81.04	80.04	10.97	2.41
U-Net + Transformer Encoder	81.21	72.82	82.19	81.72	10.25	1.94
DSAT-UNet	82.16	73.94	84.37	83.99	9.12	1.29

## Conclusion

This study introduces a novel dual Self-Attentive Transformer UNet (DSTUnet) for precise pancreatic segmentation from abdominal computed tomography (CT) images. Our model overcomes the drawbacks of conventional DCNN-based U-Net designs by successfully capturing global contextual information through the integration of twin self-attention Swin Transformer modules in both the encoder and decoder. Furthermore, the proposed DSTUnet model enhances fat fraction estimation by providing more precise and anatomically accurate pancreatic segmentation. The segmentation results were correct, underscoring the model’s ability to define pancreatic structure, with a Hausdorff Distance of 2.7 mm and a Dice Similarity Coefficient of 93.7%. The fat volume fraction (FVF) was 13.37%, and the mean pancreatic volume was 92.42 ml. These findings confirm that DSTUnet facilitates research into metabolic illnesses and supports clinical decision-making, as it accurately segments the pancreas and enhances fat estimation. In the future, multi-modal images will be investigated to improve the accuracy of pancreatic tumor segmentation. Anatomical structures and density variations can be visualized with CT, but MRI offers superior soft-tissue contrast, which is particularly beneficial in identifying tumor boundaries and heterogeneity. It is possible to extract complementary features by combining data from multiple imaging modalities (e.g., CT for structural clarity and MRI for tumor texture and borders). Additionally, features from each modality can be successfully integrated using multi-modal fusion networks, such as attention-based fusion or multi-branch encoder-decoder models. This method is expected to enhance segmentation accuracy, particularly in challenging situations where tumors are primarily visible in single-modal scans, have irregular shapes, or exhibit low contrast.

## Data Availability

The data supporting the findings of this study are publicly available online via the following links: AMOS dataset, WORD dataset, BTCV dataset, and ITK-SNAP dataset. https://www.synapse.org/Synapse:syn3193805/wiki/89480 (Date Accessed: 26 February 2025); https://jiyuanfeng.github.io/AMOS/ (Date Accessed: 12 March 2025); https://github.com/HiLab-git/WORD (Date Accessed: 7 April 2025); https://drive.google.com/drive/folders/18gBNTIMBF6xLO1exc7ISnFsN8TsFKzS8 (Date Accessed: 9 April 2025)

## Abbreviations

AI: Artificial Intelligence
ANTs: Advanced Normalization Tools
CT: Computed Tomography
DCNNs: Deep Convolutional Neural Networks
DSAT: Dual Self-Attentive Transformers
DSTUnet: Dual Self-Attentive Transformer Unet Model
DuSAL: Dual Self-Attention Layers
FCN: Fully Convolutional Networks
HU: Hounsfield Unit
IDEAL: Iterative Decomposition with Echo Asymmetry and Least-Squares Estimation
LayerNorm: Layer Normalization
MCA: Multi-Head Cross-Attention
MLP: Multilayer Perceptron
MRS: Magnetic Resonance Spectroscopy
PANDA: Pancreatic Cancer Detection with AI
R-CNN: Region-Based Convolutional Neural Network
TGPFN: Transformer-Guided Progressive Fusion Network
